# Molecular Advances in MAFLD—A Link between Sphingolipids and Extracellular Matrix in Development and Progression to Fibrosis

**DOI:** 10.3390/ijms231911380

**Published:** 2022-09-27

**Authors:** Adrian Kołakowski, Sylwia Dziemitko, Aleksandra Chmielecka, Hubert Żywno, Wiktor Bzdęga, Tomasz Charytoniuk, Adrian Chabowski, Karolina Konstantynowicz-Nowicka

**Affiliations:** 1 Department of Physiology, Medical University of Bialystok, 15-089 Bialystok, Poland; 2 Department of Ophthalmology, Antoni Jurasz University Hospital No. 1, 85-094 Bydgoszcz, Poland

**Keywords:** metabolic associated fatty liver disease, MAFLD, metabolic diseases, fatty liver, liver fibrosis, sphingolipids

## Abstract

Metabolic-Associated Fatty Liver Disease (MAFLD) is a major cause of liver diseases globally and its prevalence is expected to grow in the coming decades. The main cause of MAFLD development is changed in the composition of the extracellular matrix (ECM). Increased production of matrix molecules and inflammatory processes lead to progressive fibrosis, cirrhosis, and ultimately liver failure. In addition, increased accumulation of sphingolipids accompanied by increased expression of pro-inflammatory cytokines in the ECM is closely related to lipogenesis, MAFLD development, and its progression to fibrosis. In our work, we will summarize all information regarding the role of sphingolipids e.g., ceramide and S1P in MAFLD development. These sphingolipids seem to have the most significant effect on macrophages and, consequently, HSCs which trigger the entire cascade of overproduction matrix molecules, especially type I and III collagen, proteoglycans, elastin, and also tissue inhibitors of metalloproteinases, which as a result cause the development of liver fibrosis.

## 1. Introduction

Metabolic-Associated Fatty Liver Disease (MAFLD) could be called a silent epidemic. Today, it is a major cause of liver diseases globally, which afflict nearly 25% of the global population and its prevalence is expected to grow in the coming decades due to changes in society’s lifestyle [1,2]. This results in not only general impairment of human health but also an economic problem that burdens healthcare systems and needs to be accounted for [3].

Since 2021, scientists have recommended a change in nomenclature of non-alcoholic fatty liver disease (NAFLD) to MAFLD, which is now defined as hepatic steatosis confirmed by histology (biopsy), radiological imaging, or blood biomarker evidence of fat accumulation in the liver with the presence of one of the following criteria: overweight/obesity, type 2 diabetes mellitus (T2DM), or evidence of metabolic dysregulation. This new definition highlights the role of metabolic factors in the pathogenesis of MAFLD. Moreover, the proposed classification offers the possibility to effectively identify patients with an elevated risk of fibrosis and consequently developing progressive liver diseases, such as cirrhosis and Hepatocellular carcinoma [4]. The first complication of MAFLD is non-alcoholic steatohepatitis (NASH) understood as a state in which steatosis is combined with inflammation. The more severe liver disease being a continuum of hepatic steatosis is fibrosis. This wider perspective on fatty liver disease may allow for comprehension of the process which results in the development of advanced stages of MAFLD allowing for finding more effective personalized therapy. It seems to be even more important since not every steatosis leads to inflammation and increasing studies have shown that fibrosis may occur on the ground of steatosis with little, if any, inflammatory change. On the other hand, a disadvantage of the MAFLD definition is that it excludes a group of subjects with significant steatosis, but without metabolic risk, who previously was included in NAFLD [5].

## 2. Pathogenesis of MAFLD

The pathogenesis of steatosis in MAFLD is multicausal and is shown in Table 1. The accepted “Multiple-Hit Hypothesis” explains that fat accumulation in the liver is the result of the occurrence of multiple factors, which influence hepatic metabolism and lead to the storage of triacylglycerols (TGs) inside the hepatocytes. The main source reported as 59.0 ± 9.9% of stored fatty acid (FA) in NAFLD patients [6] arise from TGs originally released from adipose tissue to the circulation. The 26.1 ± 6.7% of TGs can also be synthesized de novo in hepatocytes during lipogenesis and 14.9 ± 7% is derived from dietary sources [6].

### 2.1. Diet

Undoubtedly, obesity and an elevated content of adipose tissue are two main risk factors for developing MAFLD [7]. Considering diet as one of the factors affecting lipid deposition, it should be remembered that not only an imbalance between energy intake and expenditure predisposes to overweight and insulin resistance, but also a type of consumed nutrition is important. The best example is the increased supply of fructose which has lipogenic potential due to the activation of transcription factors, including carbohydrate response element binding protein (ChREBP) and sterol regulatory element binding protein 1c (SREBP1c), responsible for the intensification of gluconeogenesis and de novo lipogenesis in hepatocytes [8]. Moreover, consuming fructose results in elevated plasma TGs levels [9]. Going further, diets containing saturated fatty acids (SFA) and trans-fatty acids promote steatosis [10], compared to monounsaturated fatty acids (MUFAs) and polyunsaturated fatty acids (PUFAs) which are considered to have a positive effect on the liver lipid metabolism [11].

### 2.2. Insulin Resistance

Another condition that plays a crucial role in the pathogenesis of MAFLD is insulin resistance. The reduced response of adipose tissue to the antilipolytic effect of insulin results in the generation of free fatty acids and glycerol, as a consequence of TG degradation [12]. The released into circulation free fatty acids are stored in the tissues unsuitable for this purpose such as the liver leading to steatosis development [13]. Furthermore, an elevated level of insulin also promotes hepatic de novo lipogenesis via activation of SREBP1c and ChREBP [14]. Moreover, the presence of insulin resistance also affects glucose metabolism in tissues other than the liver such as skeletal muscles which result in glucose uptake by this tissue causing hyperglycemia which as a vicious circle enhances insulin resistance [15,16].

### 2.3. Microbiota

Among factors that promote MAFLD, development should also have distinguished impairment on the gut–liver axis [17]. The modification of the intestinal microbiota induced by diet may elevate free fatty acid absorption by increasing the permeability of the intestinal wall [18]. Changes in microbiota are also associated with the elevated synthesis of short-chain fatty acids (SCFAs) [19]. SCFAs modulate lipogenesis, production of cholesterol, and glucose homeostasis; consequently, the influence of those processes yields fat accumulation in the liver (20). Moreover, dysbiosis leads to a decreased level of fasting-induced adipocyte factor (FIAF), also known as angiopoietin-related protein 4 (ANGPTL4), which inhibits the activity of lipoprotein lipase (LPL) present in the liver [20]. Consequently, increased activity of the LPL leads to enhanced release of FA from very low-density lipoprotein (VLDL) particles and higher lipid accumulation in the liver [21].

### 2.4. Genes

Recent studies revealed that genetic predisposition could also be considered an element leading to the development of steatosis. One of the examples may be a polymorphism in the patatin-like phospholipase domain containing 3 (PNPLA3) and transmembrane 6 superfamily member 2 (TM6SF2) genes that are related to an increased risk of exacerbating MAFLD occurrence [22], whereas they are not correlated with the development of coronary heart disease [23].

**Table 1:** The factors that promote MAFLD development.

Factor	Mechanism	References
**M**ETABOLIC		
**Obesity**	increased fat accumulation in the hepatocytes	[6,7]
**Western diet**	increased supply of fructose responsible for the intensification of gluconeogenesis and de novo lipogenesis in hepatocytes increased plasma TGs levels	[8,9]
**Modification in microbiota**	increased free fatty acid absorption by increased permeability of the intestinal wall increased synthesis of SCFAs that modulate lipogenesis, production of cholesterol, and glucose homeostasis decreased level of FIAF, which leads to increased activity of the LPL andincreased release of FAs from VLDL particles, and increased lipid accumulation in the liver	[18,19,20,21]
**Hypertriglyceridemia**	increased TGs accumulation in the hepatocytes	[6,13]
**Hyperinsulinemia**	promotion of hepatic de novo lipogenesis via activation of SREBP1c and ChREBP	[14]
**Insulin resistance**	generation of free FAs and glycerol resulting from TGs degradation and their storage in the liver	[12,13]
**I**NFLAMMATORY		
**HIV**	increased metabolic comorbidities hepatotoxic effect of lifelong antiretroviral therapy	[24]
**Hepatitis C infection**	the imbalance between pro-inflammatory and anti-inflammatory bioactive lipids increased ROS production increased lipid peroxidation	[25]
**Oxidative stress**	induction of hepatocytes injury by the inhibition of the mitochondrial respiratory chain enzymes increased ROS production increased lipid peroxidation increased cytokine production	[26,27]
**GENETIC**		
**Polymorphism in various genes:**		
**PNPLA3**	increased fat accumulation in the hepatocytes increased liver enzymes	[22,28]
**TM6SF2**	increased fat accumulation in the hepatocytes	[22,29]
**GCKR**	increased fat accumulation in the hepatocytes decreased β-oxidation	[29,30]

ChREBP—carbohydrate response element binding protein, FAs—fatty acids, FIAF—fasting-induced adipocyte factor, GCKR—glucokinase regulatory protein, LPL—lipoprotein lipase, PNPLA3—patatin-like phospholipase domain containing 3, SCFAs—short-chain fatty acids, SREBP1c—sterol regulatory element binding protein 1c, TGs—triacylglycerols, TM6SF2—transmembrane 6 superfamily member 2, VLDL—very low-density lipoprotein.

## 3. ECM in Healthy Liver

An extracellular matrix (ECM) is defined as a dynamic, three-dimensional scaffold composed of extracellular proteins [31]. It is now well established that ECM not only plays a crucial role in spatial support of the cells but is also engaged in liver formation and maintenance of its physiological functions. ECM interacts with cells via cell surface receptors, intracellular signaling pathways, and the release of cytokines or growth factors to maintain tissue homeostasis [32,33]. Despite the broad role of ECM in the liver, it constitutes a very limited area of healthy organ; only up to 10% of the liver volume [34] is located in the area of portal tracts, sinusoid walls, and central veins [35]. The components of the ECM and its function are presented in Table 2.

There are two main types of ECM: the interstitial matrices surrounding cells and pericellular matrices remaining in close contact with cells. Basal membrane, an example of the pericellular matrix, is composed of collagen type IV, laminins, nidogen 1 and 2 as well as proteoglycans, due to it acquiring stability and is provided with cell adhesion sites [36]. Naba et al. identified more than 150 proteins that are components of the healthy human liver ECM [37], in particular, fibrillar proteins (large number of collagens, proteoglycans, glycoproteins, including the most abundant fibrinogens and fibronectins) as well as matrisome-associated proteins (i.e., transmembrane proteoglycans, matrix metalloproteinases or cytokines) [38]. Additionally, Verstegen and collaborators presented the most abundantly occurred proteins in decellularized human livers: collagen type III, collagen type I, carbamoyl-phosphate synthase 1 (CPS1), collagen type VI, and neuroblast differentiation-associated protein (AHNAK), respectively [39]. Important ECM constituents, besides proteins, are matrix metalloproteinases (MMPs) and their tissue inhibitors—TIMPs, playing a key role in regulating ECM composition. All mentioned matrix components can be produced by hepatocytes as well as hepatic stellate cells (HSCs), Kupffer cells, and sinusoidal endothelial cells [34].

### 3.1. Collagens

The predominant structural protein in ECM is collagen, which constitutes up to 30% of total body proteins [40]. Thus far, 28 various types of collagens, encoded by more than 40 genes in the human genome, were recognized, all of them characterized by highly stabled triple helix structure [41]. Collagens can be divided into four main classes, namely: (1). fibrillar collagens (I, II, III, V, XI, XXVI, XXVII), (2). basement membrane collagens (IV, VII, XXVIII), (3). Short-chain collagens (VI, VIII, X), and (4). collagens with multiple interruptions (IX, XII, XIV, XV, XVI, XVIII, XIX–XXII) [42].

#### 3.1.1. Fibrillar Collagens

Fibrillar collagens display an important role in providing structural integrity and tensile strength, thanks to their cable-like fibril form following a wavy course in the tissue [43]. In the liver, that type of collagen is predominantly located in the portal stroma, fibroid tissue, space of Disse, and liver capsule. Moreover, fibrillar collagens are the main components of the coarse and fine reticular fibers (together with type V collagen) [44].

#### 3.1.2. Basement Membrane Collagens

Basement membrane collagens and other non-fibrillar proteins (e.g., laminin, perlecan, or nidogen) are components of a basement membrane-like matrix, which is a specialized form of ECM providing support for polarized cells but is also involved in dynamic processes such as molecules diffusion from sinusoidal blood to the liver endothelia and the opposite direction. The described structure is mainly found around the sinusoids, bile ducts as well as portal tract vessels [41,45].

#### 3.1.3. Short-Chain Collagens and FACITs

The group of short-chain collagens includes two network-forming collagens, namely type VIII and X, which display the ability to form extensive associations within the protein network [46]. The last class includes collagens with multiple interruptions containing a more specific group, namely the fibril-associated collagens with interrupted triple helices (FACITs). FACITs are associated with the surface of other collagen fibrils linking them together and with other ECM molecules, such as selected glycoproteins and proteoglycans [47,48]. This type of collagen, although present in a healthy liver, comprises the smallest number out of all the classes [38].

#### 3.1.4. Changes during Aging

It is well established that, during aging, the level of fibrous forms in ECM elevates, causing stiffness. The study showed that, in the liver of old rats (19 months), collagen deposition primarily in the periportal area of hepatic lobules was observed. The dominant form was collagen type III, rather than type I, although, in normal conditions, these two types are in approximately equal quantities [49]. Recently, Acun and collaborators performed decellularization of young and old rats and human livers. As expected, an elevated level of total collagen content, with a higher level of collagen type VI, was detected in old livers. Interestingly, the content of non-fibrillar collagens (especially type XIV and XVIII) was decreased or even absent in old liver ECM [50].

### 3.2. Glycoproteins

Glycoproteins are a large group of proteins consisting of oligosaccharide chains covalently attached to the protein core. Naba et al. identified 44 glycoproteins in liver matrisome, among which fibrinogens and fibronectins as proteins produced in the liver were found in the greatest amount. Additionally, proteins associated with elastic fiber formation, periostin as well as the tenascins C and X were the most abundant glycoproteins in the liver matrisome [37,38].

#### 3.2.1. Fibrinogen and Fibronectin

Fibrinogen is a complex α2β2γ2 hexameric glycoprotein with globular domains. In the plasma, fibrinogen is enzymatically converted to fibrin, which is an important component of thrombus, thus this protein plays a crucial role in hemostasis. However, it is also an important factor in ECM physiology due to its ability to bind to various molecules, involving growth factors, fibronectin, albumin, von Willebrand factor, or fibulin [51,52].

Fibronectin is found in the organism in two forms: plasma and cellular fibronectin, and the cellular form is less abundant in the healthy liver. In the organ, the glycoprotein is the crucial component of a membrane-like matrix, principally located in the space of Disse [53]. The master organizer, as it is called, may attach to cell surface receptors, mainly integrin and other molecules such as collagen or proteoglycans [29]. Moreover, it is a key factor in ECM formation and maturation, not to mention wound healing and embryonic development [54,55].

#### 3.2.2. Periostin, Tenascin C Nad X

Another protein associated with elastic fiber formation—periostin—was established to exert a protective effect on several tissues, thus dysfunctions in its expression are important factors in different organ disorders, including liver diseases. The protein is also engaged in cell proliferation, cancerogenesis as well as an inflammatory response [56]. Tenascins are common components of soft and connective tissues. Both tenascin C and X are engaged in tissue regeneration and recovery after mechanical injuries [36].

#### 3.2.3. Laminins

Crucial core matrisome glycoproteins are also laminins. Being important components of the basement membrane, they participate in the interactions between cells and ECM. Cell adhesion, migration, differentiation, and phenotype stability are influenced by laminins [57]. As in vitro studies demonstrated, laminins also support the proliferation and expansion of hepatic progenitor cells (HPCs) during liver damage in humans and rodents [58,59].

#### 3.2.4. Changes during Aging

Physiologically, during aging, the content of the liver’s ECM glycoproteins is increasing. The analysis demonstrated that both in old livers of humans and rodents the levels of laminins and fibrinogens were markedly heightened. However, tenascins play a significant role during tissue development, thus their content was higher in young organs.

### 3.3. Proteoglycans

Proteoglycans (PGs) are highly hydrophobic molecules with glycosaminoglycan (GAG) chains covalently attached to the protein core. The predominant group of liver ECM proteoglycans is small leucine-rich repeat proteoglycans (SLRPs), namely biglycan, decorin, asporin, or lumican [38]. All the SLRPs are enabled to interact with tyrosine kinases as well as innate immune receptors and consequently regulate different signaling pathways [60]. They regulate cell–matrix crosstalk both directly through the specific receptors and indirectly by binding to the cytokines and growth factors [61].

#### 3.3.1. Decorin

Decorin demonstrates anti-tumor properties through the direct binding to transforming growth factor-β (TGF-β) and suppressing cell growth as well as acting as a negative regulator toward hepatocyte growth factor receptor (c-Met), insulin-like growth factor receptor I (IGF-IR), vascular endothelial growth factor receptor 2 (VEGFR-2) and platelet-derived growth factor receptor (PDGFR) [62,63]. The described PG is mainly located in the area of portal tracts, central veins as well as sinusoidal walls in the healthy liver.

#### 3.3.2. Biglycan

In contrast to decorin, biglycan acts as a pro-angiogenic and pro-inflammatory factor [64]. By activating macrophage toll-like receptors (TLRs) 2 and 4, it modulates the production of cytokines that induce inflammation (i.e., tumor necrosis factor α (TNF-α) or interleukin (IL)-1β) [65,66].

#### 3.3.3. Asporin

Another member of SLRPs is asporin, a protein related to biglycan and decorin. Importantly, the molecule was proposed to act as a negative regulator to TGF-β, thus plausibly playing a role in liver disorders [64]. Generally, the content of proteoglycans in the liver matrix is elevating with age, as was presented in young livers of humans and rats, where the level of lumican was significantly lower than in old organs [50].

### 3.4. Matrix Metalloproteinases

The main proteins responsible for tissue degradation and remodeling are matrix metalloproteinases. MMPs are a family of zinc-dependent metalloendopeptidases, secreted as zymogens (pro-MMPs) that require activation. The enzymes may be divided into five main groups, distinguished by their substrate specificity: collagenases, gelatinases, membrane-type, stromelysins, and matrilysins [67]. MMPs possess the ability to degrade each component of the ECM, including the basement membrane. Besides their role in tissue rearrangement, they are also crucial mediators of physiological processes such as cell proliferation and migration, differentiation, or apoptosis [68].

In a healthy liver, the main cells responsible for MMP production are HSCs; however, hepatocytes, macrophages as well as infiltrated leukocytes also demonstrate this ability [69]. Although 23 MMPs are found in humans, only a few of them are natively expressed in the healthy liver, namely MMP-1, MMP-2, MMP-3, MMP-11 as well as MMP-13. They are presented in a small amount in normal conditions; however, their expression rapidly increases after liver injury [68,70]. Besides the already mentioned role in the degradation of matrix components, MMP-1, MMP-2 and MMP-3 are important regulators of various cytokines and chemokines via proteolytic cleavage [71]. MMP-2 is also engaged in preserving liver vascular homeostasis, mainly by mediating TGF-β activation [68].

#### 3.4.1. Tissue Inhibitors of Metalloproteinases

The activity of MMPs in tissues is precisely regulated by their inhibitors, namely tissue inhibitors of metalloproteinases (TIMPs). Any disruption in the balance between MMPs and TIMPs disturbs homeostasis and therefore is connected with various pathologies, such as tumor invasion or liver injuries [72]. Among four identified TIMPs, only TIMP-1 and TIMP-2 possess the ability to inhibit all MMPs [73]. Moreover, it was presented that TIMP-1 is involved in the promotion of survival of various cells, including HSCs or endothelial cells through inhibition of MMP activity [74]. TIMP-3 on the other hand is engaged in regulating liver lymphocyte infiltration, suggesting its role in tissue homeostasis [75].

#### 3.4.2. Changes during Aging

In aging livers, the levels of MMPs have been markedly decreased. Studies demonstrated a drop in the content of MMP-1 and MMP-2 in the livers of old rats, which led to increased collagen accumulation [49]. Additionally, the concentrations of TIMP-1 and TIMP-2 were the highest in the livers of young mice and lowered with age. Pronounced reduction of MMP activity in older organs was also confirmed in the mice model [76].

### 3.5. Cytokines and Growth Factors

ECM displays the indirect and direct paracrine role, serving as a reservoir of various cytokines and growth factors. Molecule release is precisely regulated by the matrix proteins due to wound-healing and tissue regeneration processes [34]. The main components responsible for binding molecules are SLRPs, a major group of proteoglycans, described above. They can influence the activity of various cytokines and growth factors, such as TNFα, insulin growth factor (IGF), hepatocyte growth factor (HGF), TGF-β, von Willebrand factor (VWF), and platelet-derived growth factor (PDGF), which explain their diverse function and involvement in a broad spectrum of physiological processes [77].

Proteoglycans interact with the mentioned cytokines through core proteins or GAG chains. As an example, TGF-β is known to bind to leucine-rich repeat structures, thus plausibly most SLRPs may interact with the cytokine. However, studies suggest that other proteins also specifically bind growth factors, impacting cell physiology. Notably, TGF-β is also known to attach to fibrillar proteins, such as fibronectin or fibrillins via the large latency complex (LLC). In such a complex, the cytokine is held in an inactive form until it is released by proteolytic or mechanical degradation [34]. Moreover, VEGF interacts with fibronectin and tenascin-C, resulting in the promotion of cell proliferation, whereas decorin mainly interacts with IGF, PDGF, and VWF [77,78]. Additionally, some matrix components display the ability to bind to various chemokines and cytokines infiltrated into the organ during infection to attract and activate immune cells. Fibronectin, laminin, and collagen IV have an affinity for IL-7 and IL-2, whereas TNFα primarily binds to fibronectin and laminin [79,80]. On the other hand, inflammatory cytokines, such as TNFα, TGF-β, PDGF, or IL-6, might also influence the expression and activity of matrisome components, which is especially highlighted in liver disorders [32].

#### Changes during Aging

According to the study of Acun and collaborators, the content of matrisome growth factors is decreasing in older organs. In old rat livers, the levels of fibroblast growth factor (FGF), granulocyte-macrophage colony-stimulating factor (GM-CSF), HGF, as well as VEGF were considerably lowered than in young organs, which influences liver regenerative abilities [50].

## 4. ECM in MAFLD

**Figure 1:**
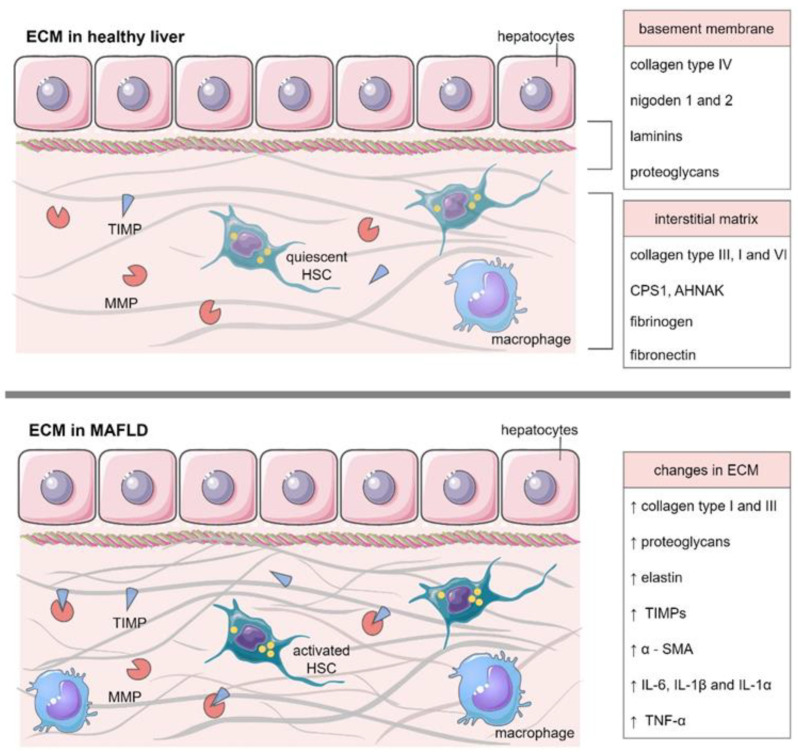
Extracellular matrix composition in healthy liver and MAFLD. α-SMA—smooth muscle alpha-actin, AHNAK—neuroblast differentiation-associated protein, CPS1—carbamoyl-phosphate synthase 1, HSC—hepatic stellate cell, IL—interleukin, MMP—matrix metalloproteinase, TIMP—tissue inhibitors of metalloproteinases, TNF-α—tumor necrosis factor α. The figure was created with aid of the Servier Medical Art.

The accumulation of the extracellular matrix and the inflammatory processes taking place therein mainly depend on the balance between ECM formation and degradation. Increased accumulation of ECM and inflammatory processes lead to progressive fibrosis, cirrhosis, and ultimately a liver failure, which is the main cause of death in MAFLD patients [81]. The Figure 1 presents changes in ECM during MAFLD development and progression.

In a healthy liver, the main cells responsible for the production of ECM are HSCs located in the perisinusoidal space (space of Disse) which are surrounded by hepatocytes and endothelial cells. HSCs are resident mesenchymal cells, retaining the characteristics of fibroblasts and pericytes, and constitute 5–10% of all resident liver cells. HSCs contain unusual components such as vitamin A, lipids, and cytoskeleton markers, and their main function is the secretion of collagens, proteoglycans, and laminin [82,83]. In response to paracrine stimulation by hepatocyte damage and macrophage activation, HSCs are transformed into a proliferative, fibrogenic, and contractile myofibroblast phenotype, showing pro-inflammatory and secretory properties [84]. HSCs can be stimulated by Kupffer cells through the action of cytokines such as transforming growth factor β1 (TGFβ1), IL-1, TNF, and reactive oxygen species (ROS) [85].

### 4.1. The Role of MMPs and TIMPs in MAFLD Deterioration

Activated HSCs overproduce matrix molecules, especially type I and III collagen, proteoglycans, elastin, and also TIMPs. TIMP-1 and -2 block MMPs such as MMP-2 and MMP-9, which are involved in inhibiting liver fibrosis. This is because MMP-2 exhibits elastase and collagenase activity and is involved in the degradation of fibrous collagens. Moreover, MMP-2 may also play a role in the degradation of mature fibrosis, consisting of collagen type I, III, and elastin. MMP-9 has been clearly shown to be of key importance in reversing liver fibrosis, but its exact mechanism of action has not yet been elucidated. [86,87]. Thus, activation of TIMPs causes an alteration of the balance between metalloproteinases and their inhibitors, resulting in the deposition and impairment of the ECM architecture [88]. However, an increase in proMMP-2, and also to a lesser extent active MMP-2, suggests a continuous rotation of the active matrix even in advanced liver fibrosis. The presence of active MMPs even in advanced stages of fibrosis may signal the possibility of reversibility of this phenomenon, which should be further investigated [87]. As a result of the deposition of ECM proteins in the Disse space, the stiffness and density of the ECM increase. The transition from type IV collagen, heparan sulfate proteoglycan, and laminin to type I and III fibrous collagen in the ECM lead to those changes and, as a result, form scar tissue [89]. A study conducted by Munsterman et al. indicated that, in patients with advanced MAFLD-associated fibrosis, elevated levels of α-SMA, proMMP-2, as well as TIMP-1 and -2 are observed [87]. Expanded ECM also binds numerous growth factors, such as HGF, FGF, or vascular endothelial growth factor (VEGF), which can be released during matrisome remodeling, thus affecting local cells. This process is tightly regulated and may be disrupted by severe tissue trauma leading to excessive production and deposition of the extracellular matrix without degradation. This abnormal healing process leads to liver fibrosis. It is also worth noting that some MAFLD patients develop fibrosis, in which we observe increased activity of immune cytokines. Some of these cytokines, such as IL-33, promote the production of IL-13 to activate stellate liver cells. Increased activation of HSCs leads to increased ECM deposition, components, and liver fibrosis [90].

The mechanism of liver fibrosis progression follows the same patterns in major liver disease etiologies (toxic, metabolic, or viral diseases), independent of the mechanisms of primary liver injury. In contrast, on a molecular basis, a complex network of fine particle-induced signaling pathways orchestrates the interaction of profibrogenic cells [91]. Depending on the etiology of the disease, we observe increased levels of other molecules and cytokines that affect profibrogenic cells. For example, in MAFLD, lipid overload occurs, leading to the accumulation of toxic intermediates in triglyceride synthesis such as saturated free fatty acids (PUFA) and their derivatives, and the accumulation of complex lipids such as sphingolipids. Thus, indicating the link between sphingolipids accumulation and changes in ECM components seems to be of prime importance and constitutes a novel way of thinking about MAFLD deterioration.

### 4.2. Importance of Sphingolipids in MAFLD Development and Deterioration

**Figure 2:**
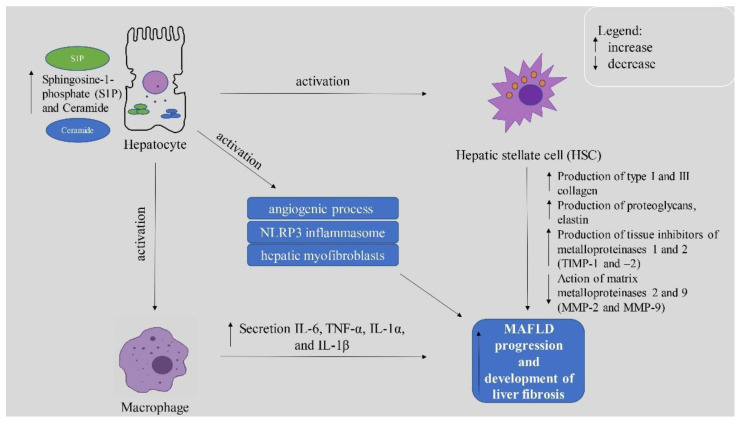
The effect of Sphingosine-1-phosphate (S1P) on the MAFLD progression to liver fibrosis. S1P: Sphingosine-1-phosphate, HSC: Hepatic stellate cell, TIMP-1 and -2: tissue inhibitors of metalloproteinases 1 and 2, MMP-2 and MMP-9: matrix metalloproteinases 2 and 9.

It is well known that increased levels of sphingolipids in humans can be observed in numerous metabolic diseases such as diabetes, obesity, and MAFLD. Increased concentration of sphingolipids in the liver is closely related to lipogenesis and the development of MAFLD and its progression to fibrosis. Increased concentration of released from hepatocytes into the ECM sphingolipids is accompanied by increased expression of pro-inflammatory cytokines, which are the main cause of disease progression to fibrosis [92]. The production of cytokines such as IL-6, TNF, IL-1β, or ROS occurs through macrophage activation by binding S1P to macrophage surface receptors, which play a key role in the progression of MAFLD to NASH [93]. The activation of macrophages is mediated by some sphingolipids whose role has not yet been fully investigated and described. In the next chapter, we will discuss the importance of the most vital sphingolipids in the development of MAFLD and progression to NASH. Moreover, the association between sphingolipids and MAFLD deterioration is presented in the Figure 2.

#### 4.2.1. Ceramide and Its Derivatives

The main sphingolipid whose increased concentration is associated with diet-dependent pathologies is ceramide. Ceramide plays a significant role also in the development and progression of liver diseases [94,95]. Yue et al. showed that HFD-rats exposed to constant light demonstrated increased accumulation of total hepatic ceramide and specific ceramide species (ceramide d18:0/24:0, ceramide d18:1/22:0, ceramide d18:1/24:0, and ceramide d18:1/24:1) [96]. The increased concentration of ceramides increases the expression of TNFα-related apoptosis-inducing ligand (TRAIL), which is responsible for the apoptosis process and the development of NASH [96,97]. However, it seems that the most important role of ceramides in the progression of MAFLD to NASH, especially hepatic C16:0 ceramide, is their presence in the production of pro-inflammatory extracellular vesicles (EVs) [98]. Under lipotoxic hepatic conditions, there is an increased accumulation of C16:0 ceramide in extracellular vesicles, which is a central element of the de novo biosynthesis pathway necessary for the release of EVs. Kakazu et al. showed that mice with early NASH exerted increased secretion of EVs enriched with high concentrations of ceramide C16:0 and sphingosine-1-phosphate (S1P). S1P is formed from sphingosine under the influence of sphingosine kinase 1 (SphK1), which is generated from ceramide thanks to the activity of ceramidase being one of the main factors activating inflammatory macrophages [99]. Another function of lipid-enriched EVs is the regulation of HSCs activation by altering the localization of microRNAs inside of the cell compartments and inhibiting PPAR-γ expression, leading to an exacerbation of fibrosis by gene expression [100]. S1P, ceramide C16:0, and EVs can be the biomarkers of early NASH and allow for early diagnosis and faster implementation of treatment [99,101].

On the other hand, inhibition of the de novo synthesis pathway or knock-down of certain ceramide synthases (CerS) reduces lipid accumulation in MAFLD [102]. Kim et al. showed increased CerS6 expression and decreased CerS2 expression in the livers of MAFLD mice fed a high-fat diet. Increased CerS6 expression resulted in increased cleavage of the sterol regulatory element-1 binding protein (SREBP-1) and enhanced lipogenesis. In contrast, artificially elevated CerS2 expression with a low dose of bortezomib inhibited the development of MAFLD [103]. Moreover, short-chain ceramides exhibit anti-inflammatory activity in various disease models. Zanieri et al. demonstrated that liposomes with C6 short-chain ceramide (Lip-C6) inhibited the proliferation of human hepatic stellate cells (hHSCs). At the same time, those liposomes caused an increase in ATP, increased activation of adenosine monophosphate kinase (AMPK), and regulation of erythroid factor 2 (Nrf2), which also indicates antioxidant and pro energetic effects of short-chain C6-ceramides [104].

We cannot define ceramides as a strictly unidirectional group. This is because long-chain ceramides are involved in the development of MAFLD, and S1P formed from ceramide may cause the progression of this disease to NASH. In contrast, short-chain ceramides inhibit lipogenesis and MAFLD development.

#### 4.2.2. Sphingosine-1-Phosphate (S1P)

Sphingosine 1-phosphate is a bioactive lipid from the sphingolipid family formed from sphingosine by phosphorylation under the influence of sphingosine kinase 1 and 2 (SPHK1 and SPHK2). The cellular levels of S1P are tightly regulated not only by SPHK but also by degradation with cleavage of the C2–C3 bond catalyzed by S1P lyase to produce hexadecenal (palmitaldehyde) and phosphoethanolamine. Moreover, specific cytosolic S1P phosphohydrolases and general cell surface lipid-phosphate phosphohydrolases (LPPs) cause dephosphorylation of S1P to sphingosine [105]. LPPs participate in cell signaling by modifying the balance between the action of lipid phosphates and their dephosphorylated S1P products. In the degradation pathway, S1P is dephosphorylated back to sphingosine by S1P phosphatases or cleaved by the S1P lyase [106].

S1P acts on cells through specific G protein-coupled receptors (S1PR1–S1PR5) that regulate cellular responses such as immunity, cellular migration, angiogenesis, and cardiac metabolism [107]. According to the latest information, S1P concentration has a significant impact on hepatic steatosis and plays the main role in the accumulation of macrophages in the liver and the development of NASH. S1P is synthesized in the liver in response to elevated lipid levels, as confirmed by numerous experiments with palmitic acid or high-fat-diet inducing steatosis in the cell and animal models, respectively [108]. Increased lipid concentration causes an elevated accumulation of ceramides undergoing the process of transformation into S1P [109]. In response to lipid overload, e.g., by palmitic acid, S1P is secreted into the extracellular matrix where it can bind to the S1PR1, S1PR2, S1PR3, and S1PR4 receptors located on the surface of macrophages and thereby stimulate receptor expression. S1P then activates HSCs, causing transdifferentiation to myofibroblastic HSCs [110].

#### 4.2.3. The Role of S1P Receptors

It is well known that macrophages induce the secretion of pro-inflammatory cytokines such as IL-6, TNF-α, IL-1α, and IL-1β, which are critical in NASH development. Liao et al. showed that, in immortalized mouse hepatocytes treated with palmitate, there is an increased release of extracellular vesicles enriched by increased concentrations of S1P, which are closely related to macrophage activity. Macrophage chemotaxis towards extracellular vesicles was significantly impaired in the case of S1P deficiency and disruption of the S1P-S1P1 signal axis, which was manifested by a reduced concentration of IL-6 [111].

It is also worth mentioning that SphK1, the enzyme that produces sphingosine-1-phosphate, requires closer investigation as there is no conclusive information about its role in fibrosis development. Geng et al. described the inducing effect of SphK1 on activation of NFκB and increased production of pro-inflammatory cytokines in obese mice. It was also shown that SphK1-null mice did not show increased symptoms of inflammation [112]. In contrast, Anderson et al. showed that SPHK1-null mice had overgrown adipocytes and reduced lipolytic activity compared to the control group. It should be noted that the levels of inflammatory cytokines and neutrophils were similar in both groups. According to Anderson et al., SphK1 has homeostatic properties in the liver and adipocytes and plays a protective role in the development of MAFLD [113]. As mentioned above, abnormal S1P signaling leads to dysfunction and fibrosis in the liver. When microcirculation is damaged, platelets are activated or thrombosis is replaced, local S1P secretion is increased in liver stellate cells, and various signaling substances are activated, such as Rho GTPase, and Hippo-Yes-associated protein (YAP) [114,115]. Liver S1P levels are closely correlated with angiogenic marker (Ang) mRNA expression, which is expressed in activated fibrotic liver HSCs and enhanced the degree of fibrosis. It follows that SphK1/S1P/S1PR1/3 plays a key role in the angiogenic process necessary for the development of fibrosis by inducing Ang1 expression through S1PR1 and S1PR3 [115].

Moreover, S1P/S1PR signaling has been shown to be involved in liver fibrogenesis by influencing the NLR family pyrin domain containing 3 (NLRP3) inflammasome, which is a critical mediator of inflammation-driven liver fibrosis. S1P was found to promote NLRP3 inflammasome stimulation and activation in a dose-dependent manner, while at the same time increased expression of SPHK1 was also observed [116]. Among the factors supporting the profibrogenic role of S1P is the effect of this molecule on hepatic myofibroblasts (hMFs). S1P had a strong migratory effect on human hMF, which showed increased expression of S1P type 1 and type 3 receptors, which resulted in their migration to damaged areas and the development of liver fibrosis [117].

The activation of HSCs and macrophages that cause fibrosis, inflammation, and the progression of MAFLD to NASH make S1P a very important factor, and it is worth treating it as a potential therapeutic target. For this reason, numerous studies have been carried out where blocking S1P and searching for substances that inhibit its action were conducted. Lu et al. described the increasing effect of amitriptyline on ceramide concentration and a de novo synthesis pathway in HFD-diet-fed mice. However, they also showed that amitriptyline reduces sphinganine and S1P production by inhibiting the ceramide hydrolysis pathway through a possible inhibition of ceramidase [118]. An S1P antagonist FTY720/fingolimod has also been shown to alleviate the symptoms of murine MAFLD and NASH by inhibiting pro-inflammatory macrophage chemotaxis. Daily administration of FTY720 reduced liver damage, inflammation, and the degree of fibrosis. Additionally, there was a decrease in the level of triglycerides and the concentration of other sphingolipids [119,120]. However, plasma levels of S1P are significantly high, which was associated with the prognosis of end-stage liver disease. Patients with the lowest levels of S1P showed the worst annual survival rate [121]. Moreover, activation of the endothelial sphingosine-1-phosphate-1 receptor (S1P1) by its natural HDL ligand (HDL-S1P) has been shown to induce liver regeneration and inhibit fibrosis. In contrast, HDL-S1P-deficient mice showed slow liver regeneration and impaired vascular remodeling [122]. Moreover, S1P is involved in sinusoidal protection against experimentally induced apoptosis and stimulates hepatocyte proliferation through IL-6 and VEGF signaling [123].

It suggests that research should be continued to investigate the role of S1P in the pathophysiology of liver disease and its potential in therapeutic interventions, as S1P is one of the most important factors in regulating the progression of NASH and the process of liver fibrosis. However, the reported results are inconclusive, which may be due to different selection patterns, and may also result from the removal of different types of liver cells, leading to different stress responses. Therefore, in future studies, more detailed experiments are needed to confirm the different roles of S1P and S1PR in the process of liver fibrosis.

## 5. Conclusions

In conclusion, the structure of the healthy liver extracellular matrix is well understood as opposed to the processes causing MAFLD and its progression to NASH. It is well known that changes in macrophages are the main factor responsible for the inflammatory response. A key regulator of macrophages, and consequently HSC function, is S1P. The gradient distribution of S1P in tissues and lymph triggers a whole cascade of events that is crucial in the development of liver fibrosis. Thus, S1P began to be treated as a new therapeutic target that would allow the inhibition of the development of fibrosis and potentially even its reversal. However, some results showed that complete removal of S1P inhibited liver regeneration. Our review provides an extensive summary of the information available to date on the role of S1P and other sphingolipids in the activation of HSCs and macrophages. It is interesting to note that there are not many reviews focusing so much on the role of sphingolipids in ECM modification. The information we collected contains all the available conclusions about the positive and negative aspects of the increased concentration of these substances in the ECM. A review of the literature showed that, despite quite unequivocal conclusions made by the authors of experimental studies, the mechanism of action of these substances should be further investigated. We demonstrate in our work that S1P may be a great therapeutic target for the treatment of MAFLD, which is intended to encourage scientists to address this topic in clinical trials.

## Figures and Tables

**Table 2:** The components and their function in healthy ECM.

ECM Components		Function
**Collagens**	1. fibrillar collagens (I, II, III, V, XI, XXVI, XXVII) 2. basement membrane collagens (IV, VII, XXVIII) 3. short-chain collagens (VI, VIII, X) 4. collagens with multiple interruptions (IX, XII, XIV, XV, XVI, XVIII, XIX–XXII)	1. providing structural integrity and tensile strength 2. providing support for polarized cells 3. forming extensive associations within the protein network 4. linking other collagens together and with other ECM molecules
**Glycoproteins**	1. fibrinogen 2. fibronectin 3. periostin 4. tenascin C and X	1. crucial role in hemostasis and binding to growth factors, fibronectin, albumin, von Willebrand factor, or fibulin 2. crucial component of a membrane-like matrix and key factor in ECM formation and maturation 3. protective effect on several tissues 4. tissue regeneration and recovery after mechanical injuries
**Proteoglycans**	1. small leucine-rich repeat proteoglycans (SLRPs) (biglycan, decorin)	1. regulation of cell–matrix crosstalk and anti-cancer effect (decorin) 2. pro-angiogenic and pro-inflammatory factor (biglycan)
**Matrix Metalloproteinases (MMPs)**	1. collagenases, gelatinases, membrane-type, stromelysins, and matrilysins 2. tissue inhibitors of metalloproteinases (TIMPs)	1. tissue degradation and remodeling; cell proliferation, migration, differentiation, or apoptosis 2. regulation of the activity of MMPs in tissues
**Cytokines and growth factors**	1. transforming growth factor beta (TGF-β) 2. tumor necrosis factor-alpha (TNF-α) 3. vascular endothelial growth factor (VEGF)	1. binding to leucine-rich repeat structures and fibrillar proteins 2. binding to fibronectin and laminin 3. interacting with fibronectin and tenascin-C, resulting in the promotion of cell proliferation

SLRPs—small leucine-rich repeat proteoglycans, TIMPs—tissue inhibitors of metalloproteinases, TGF-β—transforming growth factor beta, TNF-α—tumor necrosis factor-alpha, VEGF—vascular endothelial growth factor.

## Data Availability

Not applicable.
